# Distribution prediction of the habitat of Jingmen tick virus in China

**DOI:** 10.1128/spectrum.03430-24

**Published:** 2025-05-22

**Authors:** Weiyi Li, Rongting Li, Chengyao Liu, Jinzhi Cheng, Lin Zhan, Zhengling Shang, Jiahong Wu

**Affiliations:** 1 Key Laboratory of Modern Pathogen Biology and Characteristics, Basic Medical College, Guizhou Medical University 74628, Guiyang, Guizhou, China; 2 Department of Human Parasitology, Basic Medical College, Guizhou Medical University 74628, Guiyang, Guizhou, China; 3 School of Public Health, The Key Laboratory of Environmental Pollution Surveillance and Disease Control, Ministry of Education, Guizhou Medical University 74628, Guiyang, Guizhou, China; 4 Central Laboratory, Guizhou Provincial People’s Hospital, Guiyang, Guizhou, China; 5 Department of Immunology, Basic Medical College, Guizhou Medical University 74628, Guiyang, Guizhou, China; ^1^Changchun Veterinary Research Institute, Changchun, China

**Keywords:** jingmen tick virus, species distribution model, boosted regression trees, maxent, random forest

## Abstract

Jingmen tick virus (JMTV) is widely distributed in China, and human cases have been reported. Therefore, the objectives of this study were to understand the distribution of the JMTV in suitable areas in China under the current and projected future climates. We used two pairs of JMTV primers to detect ticks in Guizhou Province via PCR and obtained data on JMTV detection in other regions through literature research. We obtained climate data from China. Finally, maximum entropy, a boosted regression tree model, and a random forest model were used for prediction. The predictive performance of the model was evaluated by the area under the curve (AUC) of the receiver operating characteristic (ROC) curve. The JMTV positivity rate in Guizhou Province was 11.02%, which was lower than the overall national positivity rate of 16.9%. The predicted probability increased monotonically with increasing monthly mean temperature. China’s high JMTV habitat is concentrated in the Daxinganling region of Heilongjiang, Shanghai, and Hainan Provinces, with the total area of high JMTV habitat accounting for 1.94% of the country’s total land area. In the future, from 2021 to 2040, the area of JMTV habitat area in China will show an expanding trend. Our maps of JMTV distributions provide effective early warning information for monitoring JMTV and rapidly detecting outbreaks. The potential distribution of JMTV in China is expected to increase in size in the future, requiring continuous attention and surveillance.

**IMPORTANCE:**

Since the first detection of the Jingmen tick virus (JMTV) in ticks in 2014, it has been detected on several continents around the world. JMTV has also been detected in several regions of China, and human cases have been reported. JMTV has many types of hosts, including ticks, mice, bats, and turtles. It can be spread with these hosts in close proximity or over long distances. As a segmented virus, JMTV is capable not only of genetic mutation and recombination but also of genetic reassortment, resulting in changes in viral infectivity or pathogenicity. However, many uninvestigated areas still exist in China. Therefore, we investigated ticks carrying JMTV in Guizhou Province. We also predicted the distribution of JMTV in China by combining previous data.

## INTRODUCTION

Jingmen tick virus (JMTV) is an asegmented flavivirus-like positive-sense ssRNA virus that is currently classified by the International Committee on Taxonomy of Viruses (ICTV) as related unclassified viruses in the genus *Flavivirus* in the family *Flaviviridae* (1). The genomes of the JMTV contain four segments. Segment 1 encodes nonstructural protein 1, which is an RNA-dependent RNA polymerase homologous to the flavivirus NS5 protein; Segment 2 encodes nuORF and VP1, and the VP1 protein is presumed to be a viral glycoprotein; Segment 3 encodes nonstructural protein 2, which is homologous to the flavivirus NS2b-NS3 complex; and Segment 4 encodes two ORFs, a putative capsid protein and a hypothetical protein (2–4). The proteins encoded by segment 2 and segment 4 appear to be specific to this viral group.

Since the discovery of JMTV in 2014, JMTV has been found in many regions of China (5–11) and in Asia (3), Africa (12, 13), Central America (14), South America (15–17), and Europe (18, 19). Moreover, JMTV has been detected not only in ticks but also in many vertebrate hosts, such as cattle, monkeys, rodents, bats, and tortoises (7, 12). JMTV may cause human disease (9). The clinical symptoms of patients infected with JMTV vary in severity and include localized itching, headache, rash, enlarged lymph nodes, fever, and myalgia, and laboratory tests have revealed elevated concentrations of hepatic aminotransferases and slightly decreased lymphocyte counts in some patients. Some patients were admitted to the hospital with severe conditions and high fevers, and one patient even experienced a seizure. Moreover, in one study, 7 out of 10 JMTV-infected patients were also infected with skin bacteria or other viruses, with a relatively large proportion of coinfected patients (9, 20). Owing to the small sample size of JMTV patients, more intensive laboratory studies and long-term epidemiologic studies are needed to elucidate the pathogenicity of JMTV in humans. In summary, these broad geographic distributions with diverse viral hosts suggest that JMTV has become globally endemic and has the potential to cause pandemics.

At present, owing to the lack of effective drugs to combat various types of arthropod-borne infectious diseases, researchers are trying to develop long-term effective prevention and control measures by using various types of models, including infectious disease and prediction models. These models are helpful for understanding the transmission causes and outbreak cycles of infectious diseases and for predicting the possible occurrence times of infectious diseases and their epidemiological trends. Over the past few decades, many kinds of predictive models have been developed and used to forecast the effects of current and future climate change on species distributions (21). However, choosing the right model to use for individual purposes is a challenge for researchers (22). Commonly used distribution prediction models include generalized linear models (GLMs), bioclimatic models (BIOCLIM), maximum entropy models (Maxent), random forest (RF) models, and boosted regression tree (BRT) models. RF is a machine-learning algorithm based on classification trees and the Bagging algorithm (23). BRT combines boosted trees and regression trees, forming a forward selection model (24, 25). Maxent estimates the distribution of species by finding the distribution of maximum entropy under which the expected value of each environmental variable matches its empirical mean (26). Among all existing models, Maxent has the most realistic and stable predictive results, especially for endangered species, which usually have narrow distributions and better predictive performance than other models do (27).

Currently, the distribution of JMTV has been reported in some areas of China, but the distribution of JMTV in many areas of China is still unknown due to the high cost of human and material resources for sampling and detection. Therefore, the present study was designed to count the existing distribution sites of JMTV in China through literature research and self-sampling surveys. Three distribution prediction models are used to predict and draw the distribution map of JMTV in habitat areas of China and to speculate on the possible future situation of JMTV.

## MATERIALS AND METHODS

### Sample collection

We selected sampling sites in various regions of Guizhou Province, China, on the basis of the living habits of ticks, such as host preferences; single-host or multiple-host parasitism; and suitable wild habitats, such as grasslands, shrubs, and other areas of animal activity. Samples were collected from 2022 to 2023. We manually picked attached ticks from animal skins (cattle, sheep, dogs) and used cloth flags to obtain ticks from grasslands and shrubs. The ticks were quickly transported to the laboratory of Guizhou Medical University after collection. The surfaces of the ticks were disinfected with 75% ethanol and washed with deionized water. The collected ticks were initially identified morphologically, aliquoted into 1.5 mL EP tubes, and stored in a −80°C freezer until further processing. The ticks were processed by tissue grinding, the homogenate was processed by centrifugation at 10,000 × *g* for 5–10 min at 4°C, and the supernatant was transferred to a −80°C freezer for freezing and storage. Total RNA was extracted via the Qiagen Viral RNA Mini Kit. cDNA was obtained via reverse transcription of RNA samples via random primers and was subsequently purified via DNA magnetic beads. Two pairs of primers, F1/R1 and F2/R2, were used for JMTV segments 1 and 2, respectively, where primers F1/R1 are located mainly in the RDRP segment of the conserved region, and primers F2/R2 are specific to JMTV segments (Table S1). When both pairs of primers were positive, we considered the sample to be positive for JMTV.

### Literature data collection

We searched the PubMed and ZhiNET databases for published articles in English and Chinese using “Jingmen Tick Virus” as keywords. Articles that included the search terms in the title, abstract, and keywords were included in the study and subsequently screened. The literature was screened according to the following conditions: (i) Studies sampled and tested in non-Chinese regions were excluded; (ii) Chinese and English literature with duplicated content was excluded; and (iii) Studies that reached only provincial units in the collection location were excluded. Corresponding information was extracted from the retained literature: (i) the latitude and longitude coordinates of the sample collection location; (ii) the species and number of host animals of JMTV; and (iii) the number of hosts tested: if the hosts were grouped and mixed in the article, each group was counted as only one sample, and a positive test was counted as only one positive.

### Environmental data collection and filtering

Bioclimatic data were obtained from the WorldClim 2.1 database of temperature and rainfall global environmental data from 1970 to 2000, which includes 19 bioclimatic variables, such as the mean annual temperature and annual precipitation (28). Elevation data were obtained from the WorldClim 2.1 database of elevation global environmental data. We use a data set with a spatial resolution of 2.5 arcmin, which corresponds to an average grid cell size of approximately 13 km^2^. WorldClim 2.1 also provides projected future climatic information (2021–2040, 2041–2060, 2061–2080, and 2081–2100). We used the medium-resolution climate system model BCC-CSM2-MR developed by the National (Beijing) Climate Center (BCC). Considering that the WorldClim data set spans from 1970 to 2000, we also use the 1 km resolution month-by-month precipitation data set of China (1901–2022) provided by the National Tibetan Plateau Science Data Center (unit 0.1 mm) (29), the monthly mean air temperature data set at a 1 km resolution for China (1901–2022) (0.1°C) (30), the China Land Use Data Set (1980–2015) (31), and the China Regional 250 m Normalized Vegetation Index Data set (2000–2022) (32) as supplements to the biometeorological data. The population data were obtained from the LandScan Global Population Distribution data developed by the U.S. Department of Energy’s Oak Ridge National Laboratory (ORNL). The Landscan Global Population Distribution data set provides global demographic data from 2000 to 2022, with a spatial resolution of approximately 1 km × 1 km. The global demographic data of 2022 were used in this study (33).

To avoid overfitting the model due to multicollinearity between variables, it is necessary to exclude the environmental variables with higher correlations before predicting the distribution area. The “randomForest” package of R language was used to model the environmental variables 100 times to evaluate the average value of the importance of each environmental variable to the prediction model. Moreover, the correlation coefficients between the two environmental variables were obtained via correlation analysis via the R language. The environmental variables with correlation coefficients less than 0.8 were retained. If the correlation coefficient between the environmental factors was greater than 0.8, the more important environmental variables were selected and included in the subsequent analysis on the basis of the importance of the variables in the initial modeling.

### Model implementation

In this study, the “randomForest” package in R was used to implement the random forest method. Where the model parameter ntree refers to the number of trees used to build the RF, and the parameter mtry refers to the number of features considered by the decision tree when randomly selecting features. In this study, the ntree parameter is set to 1,000, and the mtry parameter is set to 5. This study uses the R language “gbm” package for BRT model construction. When constructing the model, the following parameters are set in this study: the learning rate is set to 0.01, the tree complexity is set to 5, the bag fraction is set to 70%, and the distribution type is “Bernoulli.” A randomized 30% of the data were used as the test set, and 70% of the data were used as the training set. Summarize the predicted values 1,000 times and find the average value to estimate the probability of the existence of the JMTV. In this study, Maxent (version 3.4.4) software was used to build the Maxent model, which imports only the existence of distribution data into the model. Thirty percent of the distribution points were randomly selected as the test set, and the rest were selected as the training set. The model was repeated 30 times via the cross-validation method, with the maximum number of iterations set to 5,000. The data output was in asc format, and the other parameters were set to the defaults. The average value of the 30 repetitions was taken as the prediction result.

For comparison and exploration, we used the predicted values of the three models as the covariates and whether the test was positive or not as the dependent variable and fitted a generalized linear model as a composite model to obtain the composite distribution probability (V) of JMTV. The results of the distribution probability data from the integrated model were imported into ArcGIS for the classification of suitable zones. The suitable zones of the JMTV were categorized into four types with reference to other related studies: nonsuitable zones (V ≤ 0.25), low-suitable zones (0.25 < V ≤ 0.5), medium-suitable zones (0.5 < V ≤ 0.75), and high-suitable zones (V > 0.75). The area data were also extracted via raster calculation with ArcGIS 10.7 to calculate the areas of different suitable zones, which were used to evaluate and compare the differences in the distributions of JMTVs under the current climate and the future climates of the two different models.

### Model evaluation

In this study, the area under the curve (AUC) and the accuracy of the receiver operating characteristic curve were used to validate the model. The value of the AUC ranges from 0 to 1. An AUC value greater than 0.9 indicates high accuracy, 0.8–0.9 indicates good accuracy, 0.7–0.8 indicates moderate accuracy, and less than 0.7 indicates low accuracy. The AUC value is more robust in skewed samples, and accuracy is an appropriate metric when the sample distribution is relatively balanced.

## RESULTS

### Sample collection

The literature search yielded 61 JMTV-related papers in English and Chinese, and after removing duplicates and those with noncompliant sampling points, 16 papers were included in the subsequent analysis. In this study, tick samples were collected from June 2022 to October 2023 in various cities and states in Guizhou Province; 2,160 ticks were detected, and 1,079 tick samples were tested for JMTV (Table 1). Among the 1,079 tick samples tested, a total of 199 positive samples were detected, yielding an overall positive sample rate of 11.02%.

**TABLE 1:** Summary of the JMTV sampling points used in our study

Province	Sampling point	Host	Sample size	Positive
Guizhou	Shidatou Village, Dejiang County	Rhipicephalus microplus	88	0
	Chashan Village, Zhaiying Town, Songtao County	Rhipicephalus microplus	108	17
		Hemaphysalis Iongicornis	45	0
	Tongmu Village, Shabahe Township, Songtao County	Rhipicephalus microplus	27	0
	Anhua Street, Dejiang County	Rhipicephalus microplus	14	0
	Anlong County cattle market	Rhipicephalus microplus	25	5
	Xinlongchang Township, Qianxinan Prefecture	Rhipicephalus microplus	113	57
	Boyang Township, Qianxinan Prefecture	Hemaphysalis Iongicornis	79	3
	Liupanshui Qinglin Township Ganboxu	Rhipicephalus microplus	140	35
	Mudaga Village, Panzhou City	Rhipicephalus microplus	132	0
	Cattle Market in Xiaohai Township, Weining County	Rhipicephalus microplus	42	0
	Xiongying Village, Bandi Township, Weining County	Rhipicephalus microplus	21	1
	Jinglang Village, Diping Township, Liping County	Rhipicephalus microplus	6	0
	Xinfeng Village, Diping Township, Liping County	Rhipicephalus microplus	43	0
	Tangguan Village, Caiguan Township, Anshun City	Rhipicephalus microplus	11	0
	Nucleus Village, Guangshun Township, Changshun County	Hemaphysalis Iongicornis	62	1
		Rhipicephalus microplus	21	0
	Huangtu Dapo, Maiping Town, Guiyang City	Hemaphysalis Iongicornis	16	0
	Chashan Village, Shangji Town, Zunyi City	Hemaphysalis Iongicornis	20	0
	Mugua Township, Tongzi County, Zunyi City	Hemaphysalis Iongicornis	30	0
	Longchang Township, Weining County, Bijie City	Ixodes ovatus	24	0
		Rhipicephalus microplus	12	0

We also obtained data on the detection of JMTV in other provinces of China (Table S2). After all the JMTV detection data were integrated, 3,548 pieces of detection data from 44 valid sampling sites were obtained. The total testing data included 456 bat samples, 283 rodent samples, 104 cattle samples, 26 human samples, and 2,679 tick samples, with a total positive rate of 16.9% for JMTV.

### Environmental data

All the environmental variables were screened for correlation analysis and model significance (Fig. S1 and S2). Nineteen environmental variables were ultimately retained for subsequent analysis (Table S3).

**Fig 1:**
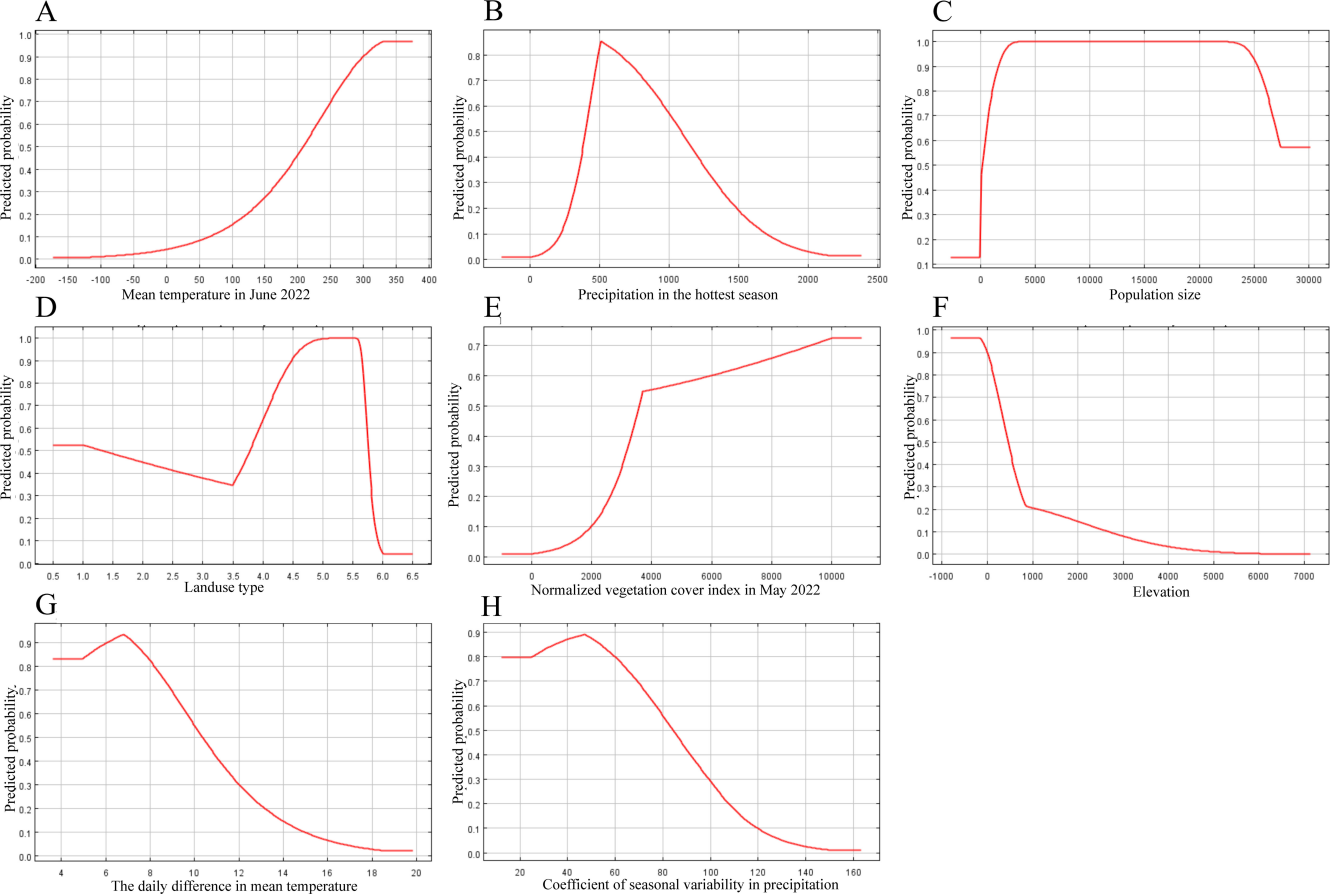
Response curves of the effects of environmental factors on the distribution of JMTV. (A) Mean temperature in June 2022; (B) precipitation in the hottest season; (C) population size; (D) land use type; (E) normalized vegetation cover index in May 2022; (F) elevation; (G) the daily difference in mean temperature; and (H) coefficient of seasonal variability in precipitation. The horizontal coordinate is the corresponding environmental variable, and the vertical coordinate is the predicted probability of the JMTV.

We also modeled the environmental variables as one-way factors and plotted response curves to understand the relationships between the distributions of the JMTV fitness zones and the corresponding single environmental variables, as shown in Fig. 1, where we illustrate eight representative environmental variables. Among them, the predicted probability monotonically increases with increasing temperature and reaches the highest probability at approximately 32.5°C; the predicted probability increases and then decreases with increasing precipitation and reaches the highest probability when the precipitation reaches 500 mm in the hottest season. Fig. 1D shows the land cover type when the land cover type is urban land, rural settlements, or other construction land, which is suitable for the existence of the JMTV. The probability of the existence of the JMTV decreases rapidly with increasing elevation to a lower value before the elevation of approximately 900 m, and the influence of elevation on the probability of the existence of the JMTV is weaker, and the probability decreasing trend slows down above 900 m.

### Model implementation

**Fig 2:**
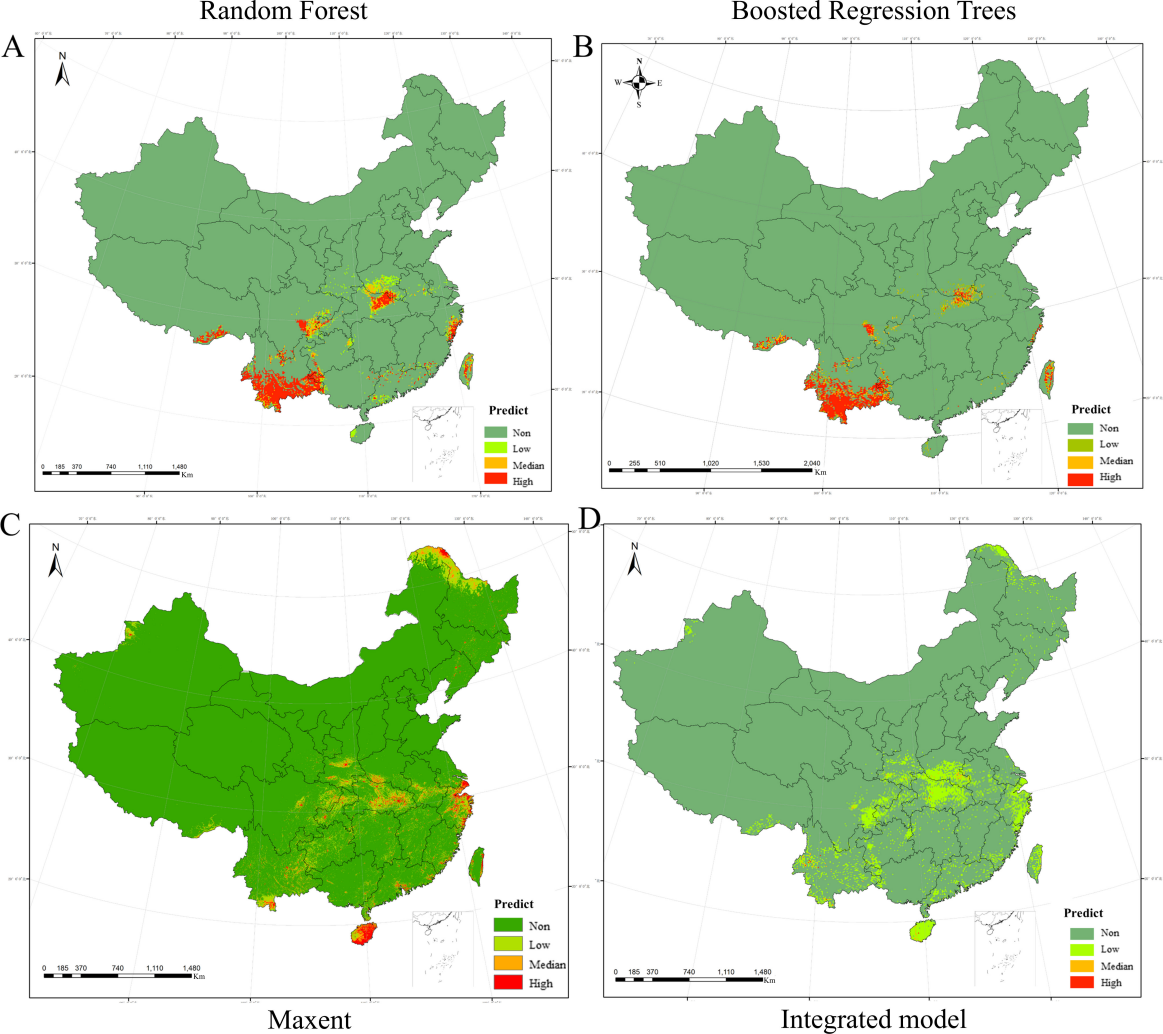
Predicting the distribution of JMTV habitat for all samples. (A). Random forest model; (B) boosted regression tree model; (C) maxent model; (D) integrated model. The cartographic foundation was established using China’s Standard Map (Approval No.: GS(2024)0650), acquired through the National Platform for Common Geospatial Information Services (Tianditu, https://www.tianditu.gov.cn/) administered by the Ministry of Natural Resources; the base map has not been modified.

As shown in Fig. 2 (and a separate Chinese map with province names in Fig. S3), when we constructed the model using all the samples, the accuracies of the three models RF, BRT, and Maxent were 77.9%, 73.0%, and 76.7%, respectively, with AUC values of 0.780, 0.772, and 0.952. The corrected *R*^2^ value of the combined model was 0.502, with an accuracy of 56.1%. The RF and BRT models predicted highly habitable zones mainly in southern Tibet, most of southern Yunnan Province, the border of Yunnan, Guizhou and Guangxi Provinces, the western part of the Sichuan Basin, the central part of Hubei Province, the southern part of Henan Province, and the south-eastern part of Zhejiang Province, with total areas of highly habitable zones of 1.67 × 10^5^ km^2^ and 2.4 × 10^5^ km^2^, respectively. Maxent predicts that the highly habitable areas are mainly concentrated in the Daxinganling area in northeast China, southern Yunnan Province, central Hubei Province and southern Henan Province, Zhejiang Province, and Hainan Province, with a total area of 3.27 × 104 km² of highly habitable areas.

**Fig 3:**
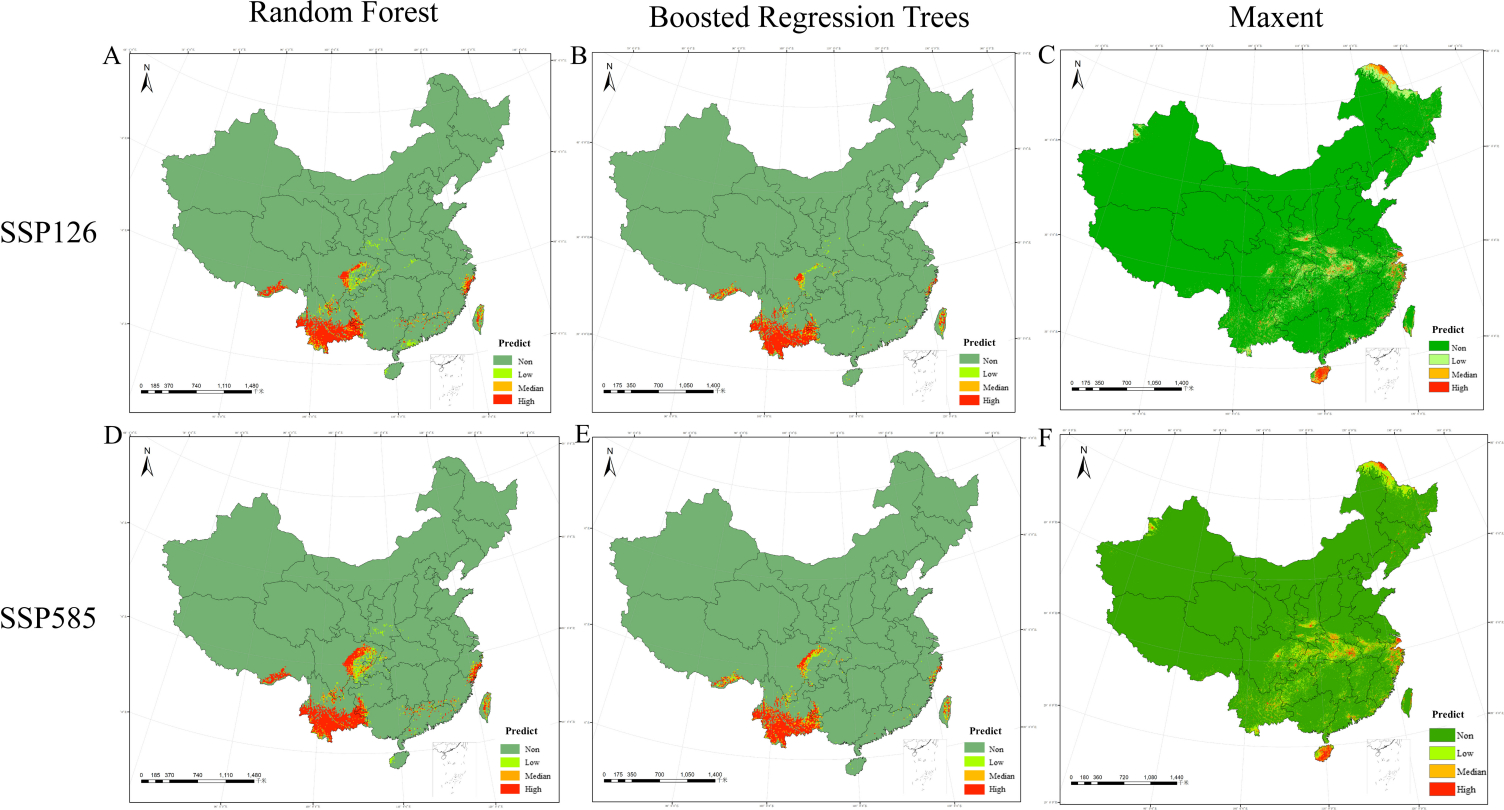
Predicting the distribution of JMTV habitat under different climate scenarios for all samples. (A) Random forest model under the SSP126 scenario climate; (B) Boosted regression trees under the SSP126 scenario climate; (C) maxent under the SSP126 scenario climate; (D) random forest model under the SSP585 scenario climate; (E) boosted regression trees under the SSP585 scenario climate; (F) maxent under the SSP585 scenario climate. The cartographic foundation was established using China’s Standard Map (Approval No.: GS(2024)0650), acquired through the National Platform for Common Geospatial Information Services (Tianditu, https://www.tianditu.gov.cn/) administered by the Ministry of Natural Resources; the base map has not been modified.

We also projected the distribution of the JMTV under two future climate scenarios for 2021–2040, as shown in Fig. 3. The future distribution projections are similar to the current distribution projections, with the RF, BRT, and Maxent models having high habitable areas of 2.63 × 10^5^ km^2^, 2.202 × 10^5^ km^2^, and 6.95 × 10^4^ km^2^, respectively, for the SSP126 scenario. The highly habitable zones under the SSP585 scenario are 2.97 × 10^5^ km^2^, 2.107 × 10^5^ km^2^, and 8.19 × 10^4^ km^2^, respectively. Overall, the size of the habitable area of the JMTV will expand in the future.

## DISCUSSION

In this study, we investigated JMTV carriage by ticks in Guizhou Province and further mapped the predicted ecological niche of JMTV in China. JMTV positivity in Guizhou Province is lower than the overall JMTV positivity rate in China. There are large and concentrated areas of high habitability in the Heilongjiang Daxinganling region, Shanghai Province, and Hainan Province, whereas the remaining areas of high habitability are more discrete, with the total area of fitness showing a trend of expansion in the future. Our prediction map can provide a scientific reference for the monitoring, prevention, and control of JMTV in China.

The results of the screening and analysis of the environmental variables revealed that the main climatic factor influencing the geographic distribution of the JMTV was the minimum temperature of the coldest month, and the probability of the presence of the JMTV increased with increasing temperature. Among the hosts of JMTV, mammals are less sensitive to climatic conditions than ticks, so the relationship between the predicted probability and temperature is similar to the relationship between ticks and temperature. Tick growth and development are influenced by temperature (34). Humidity is another important factor, and the optimum humidity for JMTV is when the precipitation in the hottest season is approximately 500 mm. Relevant studies have shown that at a constant temperature of 26°C when the relative humidity is lower than 70%–80%, the development of tick embryos is slow or stops; when the relative humidity is reduced from 100% to 50%, the hatching rate of larvae may decrease by more than 37% due to severe dehydration of eggs; and when the humidity drops to 30% or lower, all eggs cannot hatch (35–37). Although ticks are specialized blood-sucking extracorporeal parasitic vectors, more than two-thirds of their life cycle is in the nonparasitic stage, and they exist in nature as free ticks (38). Therefore, changes in temperature and humidity have important effects on tick life history and indirectly affect the distribution and spread of JMTV (39).

Similar to previous prediction studies, we did not predict the same results using the three methods, although several models performed well in general, with Maxent’s results being the most realistic in terms of AUC values. The prediction results of Maxent differ significantly from those of RF and BRT. This divergence likely stems from fundamental differences in their underlying methodologies. Maxent employs a presence-only approach that avoids subjective assumptions about unknown conditions (26). This characteristic makes it particularly advantageous for modeling rare species distributions with limited occurrence data. In contrast, both RF and BRT represent extensions of decision tree-based algorithms that require explicit presence-absence data for sample classification (23–25). This dependency makes their predictive outputs more sensitive to data composition characteristics. The information on dominant tick species may lead to RF and BRT prediction results being more biased toward predicting the distribution of dominant tick species. However, there was not much difference between the three models in terms of accuracy, which were all slightly above 80%, so we could not ignore the prediction results of the RF and the BRT. Therefore, the critical challenge lies in synthesizing these complementary predictions to formulate robust conclusions. Moreover, the maximum entropy model has a high AUC value and relatively low accuracy, which may be because our study sample is not sufficiently balanced. The AUC value is more robust than the accuracy when the sample is not balanced, suggesting that the model evaluation prefers the results of the AUC value. Additionally, the presence of high AUC values for the maximum entropy model suggests a risk of overfitting, which may ignore the potential distribution of JMTVs in other regions.

In summary, not only is there a wide range of highly habitable areas in Heilongjiang Province, but corresponding cases have also been reported (9). Previous investigations of ticks and tick-borne pathogens, which are major natural sources of tick-borne diseases in Heilongjiang Province, have revealed the presence of a variety of ticks and tick-borne pathogens in the region (6). These findings are undoubtedly not indicative of the need to continue strengthening relevant surveillance and disease prevention and control in the region to prevent possible epidemics of tick-borne diseases. Hainan Province had similar results to Heilongjiang Province, but no corresponding cases have been reported yet. Previous studies have shown that a wide range of ticks are also present in Hainan, and ticks in the region also carry a variety of viruses that are potentially pathogenic to humans (40, 41). Relevant departments need to pay attention to strengthening the management of people engaged in occupations with close contact with animals, learning and popularizing relevant knowledge, and raising awareness of prevention. Moreover, relevant monitoring should be strengthened to decrease the prevalence of JMTV. In addition, for areas where partial testing has been done, such as Hubei, Zhejiang, Yunnan, Sichuan, and Guizhou Provinces, there is already a large range of high habitability areas. Although these areas are not sufficiently concentrated, the overall area is expanding, and relevant departments need to be vigilant. For example, in Sichuan Province, only the Wolong Nature Reserve has been tested (42), and the predicted results suggest that the JMTV habitability area is expanding internally along the margins of the Sichuan Basin. Measures need to be taken in these areas to curb the expansion of the habitability area. Guangxi, Guangdong, Anhui, Jiangxi, Hunan, and Shandong are characterized mainly by sporadic dispersal of high-habitability areas, consistent with previous studies not included in the model, such as the low tick JMTV positivity rates detected in Shandong and Anhui, which were 0.7% and 3.2%, respectively (11). However, these areas need to be aware of the increase in the size of local high-habitability areas as climate change occurs in the future. More aggregated habitability areas exist in both Xinjiang and Tibet, but there have been no positive reports of ticks carrying JMTV, and testing for JMTV in rodents has been reported only in Xinjiang (5).

As future conditions change, the extent of areas with high JMTV habitability gradually increases. This finding indicates that climate change due to high carbon dioxide emissions positively affects JMTV infections and that future habitability areas are clearly spreading to neighboring areas. JMTV, as a tick-borne virus, is most at risk of infection in people who live or work near animals, and care should be taken to take appropriate measures to prevent tick bites. Moreover, the results suggest that JMTV may have a relatively large potential habitat, as it is currently relatively rare in mammals, but JMTV has been detected in rodents in northwestern Xinjiang and eastern coastal Zhejiang. We recommend monitoring infection levels of JMTV in different species as soon as possible, determining the specifics of transmission, and proactively developing intervention programs.

Our maps of the JMTV distribution provide effective early warning information for monitoring JMTV and rapid detection of outbreaks and help to avoid blind investigations, thereby saving time and human resources. It also provides opportunities for rapid and effective prevention measures and helps decision-makers optimize the allocation of control resources, thereby better targeting the comprehensive prevention and control of tick-borne diseases in high-risk areas. Moreover, this study provides scientific support for the prevention and control of infectious diseases and potential epidemics in densely populated areas of livestock in the future.

### Conclusions

JMTV positivity in Guizhou Province is lower than the overall JMTV positivity rate in China. The potential distribution area of the JMTV in China is concentrated mainly in the Daxinganling region of Heilongjiang Province and Hainan Province, but the JMTV habitat area is expected to expand in the future. However, despite the consideration of multiple modeling approaches, the utility of the results needs to be verified with more survey data.

## Data Availability

The JMTV data and tick data used in this article are available in Figshare at https://doi.org/10.6084/m9.figshare.28416860.v2.

## SUPPLEMENTAL MATERIAL

Supplemental tables and figures spectrum.03430-24-s0001.docx Tables S1 to S3 and Figures S1 to S3.

ASM does not own the copyrights to Supplemental Material that may be linked to, or accessed through, an article. The authors have granted ASM a non-exclusive, world-wide license to publish the Supplemental Material files. Please contact the corresponding author directly for reuse.
