# Supplementary material for: Distribution prediction of the habitat of Jingmen tick virus in China
Source: Microbiol Spectr. 2025 May 22;13(7):e03430-24. doi: 10.1128/spectrum.03430-24 (PMC12211064; doi:10.1128/spectrum.03430-24)
Supplement: Supplemental tables and figures — Tables S1 to S3 and Figures S1 to S3. [file spectrum.03430-24-s0001.docx]

**Supplementary Table 1** Amplification primers and sequences

| Primer | Sequence (5’-3’) | Size (bp) |
| --- | --- | --- |
| F1 | ATA GGC TGT CCAACA CCG TGA T | 1300 |
| R1 | TGG ATC TCA TTG CCG TAC TTC AC |  |
| F2 | CAAGTGCATACATCGCCAAC | 891 |
| R2 | CAAGTGCATACATCGCCAAC |  |

**Supplementary Table 2** Summary of the JMTV sampling point in other studies

| Province/autonomous | Year | Sampling point | Host | Sample size | Positive | References |
| --- | --- | --- | --- | --- | --- | --- |
| Sichuan | 2020 | Wolong nature reserve | Ixodes acuttarsus | 4 | 1 | (1) |
|  |  |  | Ixodes ovatus | 7 | 1 |  |
|  |  |  | Haemaphysalis Iongicornis | 4 | 2 |  |
|  |  |  |  |  |  |  |
| Fujian | 2019 | Legal hunting grounds in Nanping | Amablyomma testudinarium | 26 | 9 | (2) |
|  |  |  | Haemaphysalis Iongicornis | 9 | 2 |  |
|  |  |  |  |  |  |  |
| Zhejiang | 2012-2016/2010 | Wenzhou | Haemaphysalis hystricis | 102 | 47 | (3)/(4) |
|  |  |  | Ixodes granulatus | 3 | 1 |  |
|  |  |  | Rhipicephalus microplus | 113 | 60 |  |
|  | 2014/2010 |  | Cattle | 104 | 10 |  |
|  | 2016 |  | Bat | 216 | 19 |  |
|  | 2016 |  | Rodent | 119 | 8 |  |
|  | 2010 | Ningbo | Rhipicephalus sanguineus | 3 | 1 | (4) |
|  |  |  |  |  |  |  |
| Yunnan | 2015-2017 | Honghe county | Haemaphysalis Iongicornis | 95 | 10 | (5) |
|  |  | Nujiang Lisu autonomous prefecture | Rhipicephalus microplus | 77 | 0 |  |
|  |  | Luxi county | Rhipicephalus microplus | 172 | 1 |  |
|  |  | Dali Bai autonomous prefecture | Rhipicephalus microplus | 351 | 0 |  |
|  |  |  |  |  |  |  |
| Hubei | 2010 | Jingmen | Rhipicephalus microplus | 31 | 18 | (4) |
|  |  | Wuhan | Rhipicephalus microplus | 25 | 16 |  |
|  |  |  | Haemaphysalis Iongicornis | 91 | 50 |  |
|  |  |  | Ixodes sinensis | 1 | 1 |  |
|  |  | Shiyan | Rhipicephalus microplus | 25 | 16 |  |
|  |  | Shennongjialin | Rhipicephalus microplus | 25 | 16 |  |
|  |  | Xiangyang | Rhipicephalus microplus | 25 | 0 |  |
|  |  |  | Haemaphysalis flava | 15 | 2 |  |
|  |  | Xinzhou district of Wuhan | Haemaphysalis campanulata | 24 | 18 |  |
|  |  |  | Haemaphysalis flava | 15 | 1 |  |
|  |  | Huanggang | Haemaphysalis flava | 15 | 2 |  |
|  |  |  | Haemaphysalis hystricis | 32 | 0 |  |
|  |  | Yichang | Rhipicephalus microplus | 25 | 16 |  |
|  |  | Enshi Tujia and Miao autonomous prefecture | Rhipicephalus microplus | 25 | 16 |  |
|  |  |  |  |  |  |  |
| Heilongjiang | 2010-2018/- | Mudanjiang Forestry Center Hospital | Human | 26 | 14 | (6)/(7) |
|  | 2015-2016 | Amur Township, Daxinganling | Ixodes persulcatus | 11 | 3 | (8) |
|  |  | Arctic Village in Daxinganling | Ixodes persulcatus | 28 | 2 |  |
|  |  | Chona River Nature Reserve | Haemaphysalis Iongicornis | 19 | 2 |  |
|  |  |  | Haemaphysalis concinna | 25 | 1 |  |
|  |  |  | Dermacentor nuttalli | 2 | 2 |  |
|  |  |  | Ixodes persulcatus | 7 | 3 |  |
|  |  | Shuanghe Nature Reserve | Dermacentor nuttalli | 2 | 1 |  |
|  |  |  | Dermacentor silvarum | 2 | 0 |  |
|  |  |  | Ixodes persulcatus | 1 | 0 |  |
|  |  |  |  |  |  |  |
| Hainan | 2020 | Danzhou Xiqing Farm | Rhipicephalus sanguineus | 15 | 9 | (9) |
|  |  | Nankun Township, Tunchang County | Rhipicephalus sanguineus | 21 | 9 |  |
|  |  |  | Rhipicephalus microplus | 9 | 6 |  |
|  |  | Jinmo Village, Lingao County | Rhipicephalus sanguineus | 9 | 6 |  |
|  |  | Luotan Village, Qiongzhong County | Rhipicephalus sanguineus | 3 | 3 |  |
|  |  |  |  |  |  |  |
| Xinjiang | 2016 | Qabqar Siberian autonomous county | Rodent | 164 | 42 | (10) |
|  |  |  |  |  |  |  |
| Neimenggu | 2019 | Aershan Port | Dermacentor silvarum | 106 | 0 | (11) |
|  |  |  |  |  |  |  |
| Guizhou | 2012 | Anlong county | Bat | 120 | 18 | (3) |
|  |  |  |  |  |  |  |
| Henan | 2012 | Neixiang county | Bat | 120 | 17 | (3) |

**Supplementary Table 3** Environmental variables used in species distribution model.

| **NO.** | **Variable** | **Explanatory variable** |
| --- | --- | --- |
| 1 | elve | Elevation |
| 2 | HXPT_FVC_4 | Normalized Vegetation Index May 2022 |
| 3 | landscan_g | Population |
| 4 | Landuse201 | Land use type in 2015 |
| 5 | pre_202210 | Average precipitation for October 2022 |
| 6 | pre_20224 | Average precipitation for April 2022 |
| 7 | pre_20225 | Average precipitation for May 2022 |
| 8 | pre_20226 | Average precipitation for June 2022 |
| 9 | pre_20227 | Average precipitation for July 2022 |
| 10 | pre_20228 | Average precipitation for August 2022 |
| 11 | pre_20229 | Average precipitation for September 2022 |
| 12 | pre_Layer1 | Average precipitation for January 2022 |
| 13 | tmp_20226 | Average temperature in June 2022 |
| 14 | wc2_1_2__2 | Average daily difference in temperature |
| 15 | wc2_1_2__6 | Minimum temperature of the coldest month |
| 16 | wc2_1_2__8 | Mean temperature of the wettest quarter |
| 17 | wc2_1_2_15 | Coefficient of seasonal variability of precipitation |
| 18 | wc2_1_2_18 | Precipitation in the hottest season |
| 19 | wc2_1_2_19 | Precipitation in the coldest season |


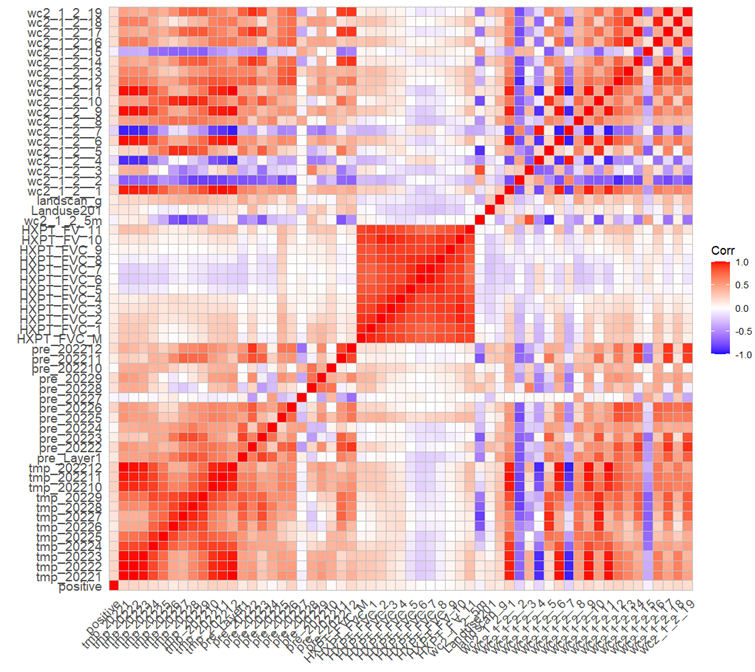


**Supplementary Figure 1** Spearman's correlation of 58 Environmental Variables


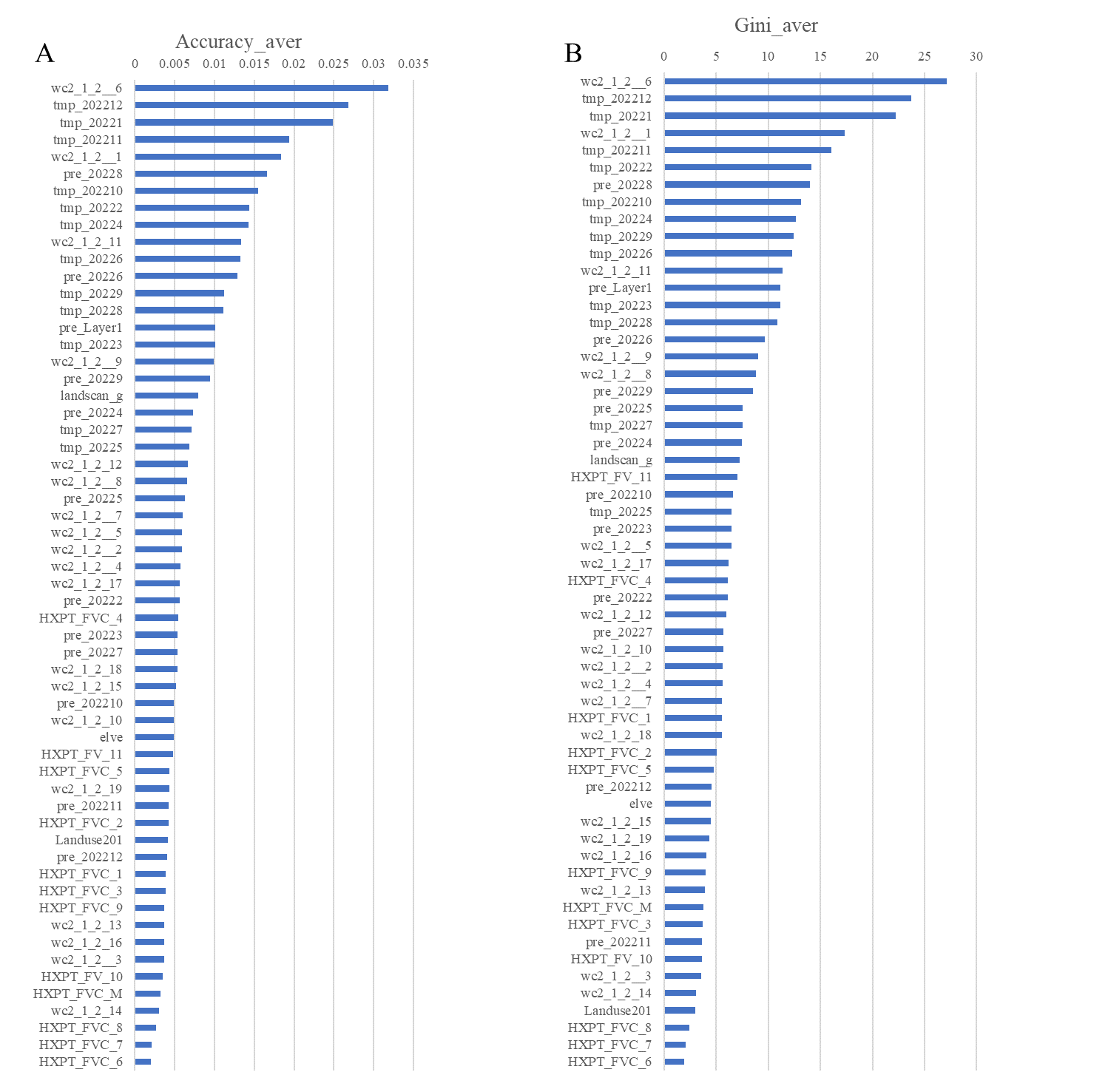


**Supplementary Figure 2** Importance of 58 environmental variables. A, average accuracy reduced when the variable is missing; B, average Gini coefficient reduced when the variable is missing.


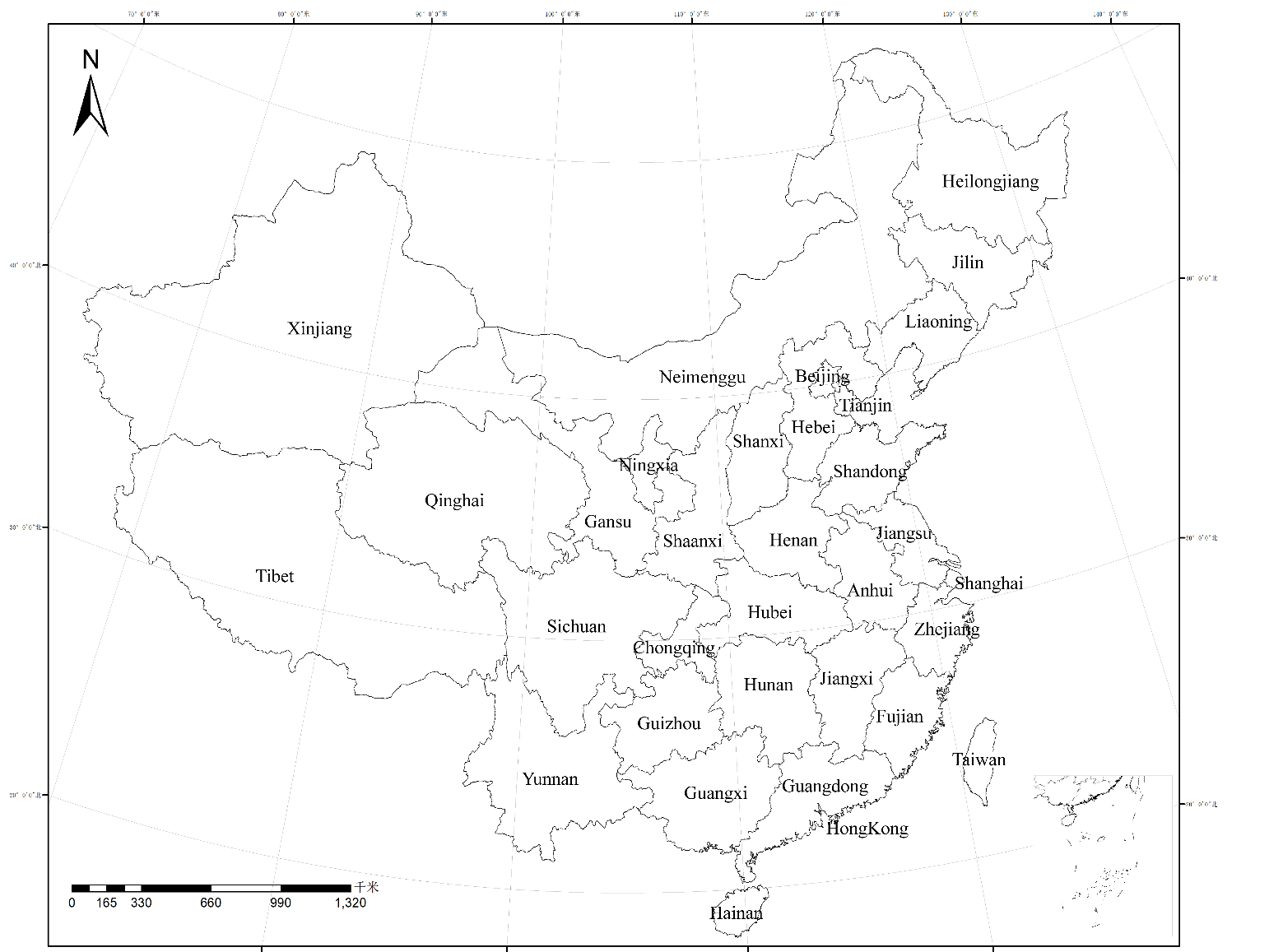


**Supplementary Figure 3** Chinese map with province names.

**REFERENCES**

1. Huang L, Liu S, Chen L, Wang F, Ye P, Xia L, Jiang B, Tang H, Zhang Q, Ruan X, Chen W, Jiang J. 2023. Identification of novel Jingmen tick virus from parasitic ticks fed on a giant panda and goats in Sichuan Province, southwestern China. Front Microbiol 14:1179173.

2. Zhang Y, Li Z, Pang Z, Wu Z, Lin Z, Niu G. 2022. Identification of Jingmen tick virus (JMTV) in Amblyomma testudinarium from Fujian Province, southeastern China. Parasit Vectors 15:339.

3. Guo JJ, Lin XD, Chen YM, Hao ZY, Wang ZX, Yu ZM, Lu M, Li K, Qin XC, Wang W, Holmes EC, Hou W, Zhang YZ. 2020. Diversity and circulation of Jingmen tick virus in ticks and mammals. Virus Evol 6:veaa051.

4. Qin XC, Shi M, Tian JH, Lin XD, Gao DY, He JR, Wang JB, Li CX, Kang YJ, Yu B, Zhou DJ, Xu J, Plyusnin A, Holmes EC, Zhang YZ. 2014. A tick-borne segmented RNA virus contains genome segments derived from unsegmented viral ancestors. Proc Natl Acad Sci U S A 111:6744-9.

5. Shi J, Shen S, Wu H, Zhang Y, Deng F. 2021. Metagenomic Profiling of Viruses Associated with Rhipicephalus microplus Ticks in Yunnan Province, China. Virol Sin 36:623-635.

6. Jia N, Liu HB, Ni XB, Bell-Sakyi L, Zheng YC, Song JL, Li J, Jiang BG, Wang Q, Sun Y, Wei R, Yuan TT, Xia LY, Chu YL, Wei W, Li LF, Ye JL, Lv QY, Cui XM, Guan Y, Tong YG, Jiang JF, Lam TT, Cao WC. 2019. Emergence of human infection with Jingmen tick virus in China: A retrospective study. EBioMedicine 43:317-324.

7. Zhang J, Zheng YC, Chu YL, Cui XM, Wei R, Bian C, Liu HB, Yao NN, Jiang RR, Huo QB, Yuan TT, Li J, Zhao L, Li LF, Wang Q, Wei W, Zhu JG, Chen MC, Gao Y, Wang F, Ye JL, Song JL, Jiang JF, Lam TT, Ni XB, Jia N. 2023. Skin infectome of patients with a tick bite history. Front Cell Infect Microbiol 13:1113992.

8. Meng F, Ding M, Tan Z, Zhao Z, Xu L, Wu J, He B, Tu C. 2019. Virome analysis of tick-borne viruses in Heilongjiang Province, China. Ticks Tick Borne Dis 10:412-420.

9. Wang G, Tian X, Peng R, Huang Y, Li Y, Li Z, Hu X, Luo Z, Zhang Y, Cui X, Niu L, Lu G, Yang F, Gao L, Chan JF, Jin Q, Yin F, Tang C, Ren Y, Du J. 2024. Genomic and phylogenetic profiling of RNA of tick-borne arboviruses in Hainan Island, China. Microbes Infect 26:105218.

10. Yu ZM, Chen JT, Qin J, Guo JJ, Li K, Xu QY, Wang W, Lu M, Qin XC, Zhang YZ. 2020. Identification and characterization of Jingmen tick virus in rodents from Xinjiang, China. Infect Genet Evol 84:104411.

11. Li LF, Zhang MZ, Zhu JG, Cui XM, Zhang CF, Niu TY, Li J, Sun Y, Wei W, Liu HB, Yuan TT, Wei R, Wang Q, Xia LY, Zhao L, Lesley BS, Jiang BG, Jiang JF, Frans J, Jia N, Cao WC. 2022. Dermacentor silvarum, a Medically Important Tick, May Not Be a Competent Vector to Transmit Jingmen Tick Virus. Vector Borne Zoonotic Dis 22:402-407.
